# A multi-modal co-attention model for accurate drug-target interaction prediction

**DOI:** 10.1371/journal.pone.0351880

**Published:** 2026-06-22

**Authors:** Wanjun Ma, Wenjun Li, Mengyun Yang, Zhengdong Pu, Xiwei Tang

**Affiliations:** 1 School of Computer Science, Hunan First Normal University, Changsha, Hunan, China; 2 Hunan Provincial Key Laboratory of Intelligent Processing of Big Data on Transportation, Changsha University of Science and Technology, Changsha, Hunan, China; Xinjiang Technical Institute of Physics and Chemistry, CHINA

## Abstract

Accurate prediction of drug-target interactions (DTIs) plays a crucial role in modern drug discovery and repositioning. Despite recent advances in deep learning, existing methods often fail to effectively integrate heterogeneous data, such as molecular structures and protein sequences, into a unified representation. To address this limitation, we propose MMCA (Multi-Modal Co-Attention), a novel deep learning framework that introduces a multi-modal co-attention mechanism to dynamically align and fuse graph-based drug features with sequence-based protein embeddings. Our model leverages parallel encoding pathways to capture both structural and semantic information, followed by a context-aware fusion module that adaptively weighs cross-modal dependencies. Evaluation on three benchmark datasets—BioSNAP, BindingDB, and Human STRING—demonstrates that MMCA outperforms state-of-the-art methods in terms of AUC, AUPR, and F1-score, achieving up to 98.4% AUC. Ablation studies confirm the significance of our co-attention fusion mechanism in enhancing both accuracy and robustness. Case studies of high-confidence predictions reveal biologically plausible drug-protein interactions, supporting MMCA’s potential for prioritizing candidates for experimental validation. By enabling end-to-end multi-modal reasoning, MMCA provides a powerful framework for advancing DTI prediction systems and offers broad applicability for various bioinformatics tasks. The source code of MMCA is publicly available at https://github.com/Join-xiaobai/MMCA.

## Introduction

Drug repositioning is a significant area of research in the biomedical field [1]. Verifying the interaction between a drug and its specific target is a crucial step in this process, as it confirms the drug’s efficacy [2]. Although traditional in vitro screening experiments are a feasible method, they have notable limitations, including high labor costs, substantial financial investment, and prolonged timeframes [3]. Additionally, drug-target interaction (DTI) prediction is essential in drug discovery, as it helps identify a drug’s potential therapeutic effects on a biological target, thus aiding in the development of effective therapeutics [4]. DTI prediction relies on detailed interaction data between a drug’s molecular structure and the key amino acids of the target protein. Therefore, accurately capturing this intricate interaction information is vital for constructing effective DTI prediction models. To enhance the efficiency of drug-target interaction assessments, DTI prediction through computer simulation has emerged as a promising alternative strategy that is gaining increasing attention and research focus [5].

The existing prediction methods can be broadly categorized into three types: ligand-based [6], structure-based [7], and machine learning-based methods [8–10]. In contrast, traditional structure-based and ligand-based electronic virtual screening methods have garnered increasing attention and usage due to their relative effectiveness [11–13]. However, these traditional approaches also have notable shortcomings. For example, the commonly used molecular docking technique is often inefficient, exhibiting slowness due to substantial computational demands, and the accuracy of its scoring function is limited [14]. Additionally, ligand-based methods are restricted in their scope and performance by the finite number of known ligands linked to a specific protein. The absence of three-dimensional structural and ligand information further hampers the advancement of structure-based methods.

As a subset of machine learning, deep learning has significantly advanced the field of drug-target interaction (DTI) prediction. In the early stages of research, scientists utilized manually designed drug and protein descriptors to make predictions using fully connected neural networks [15]. Subsequently, Lee et al. introduced the DeepConv-DTI [16] model, which employs a convolutional neural network (CNN) to extract protein features, an extended connectivity fingerprint (ECFP) [17] to calculate drug features, and a fully connected network (FCN) for DTI prediction. However, this model does not account for the interactions between drug-protein pairs.

In response, subsequent researchers have proposed more sophisticated feature extraction techniques to better capture these interactions. For instance, the dyCNN module used in SAG-DTA [18] and DrugVQA [19] illustrates ongoing efforts to enhance feature extraction methodologies.

In recent years, Graph Neural Networks (GNNs) have demonstrated significant effectiveness in key prediction tasks within bioinformatics, primarily due to their robust feature representation learning capabilities [20–22]. To extract topological information about drugs, Nguyen et al. proposed the GraphDTA model [23], which treats drugs as molecular graphs. This model employs GNNs and CNNs to extract features from drugs and proteins, respectively, and subsequently predicts their affinity. However, despite the advanced feature extraction module utilized by GraphDTA, it overlooks a critical aspect: the interaction between molecules is fundamentally based on the related substructures of drugs and proteins. Additionally, the model fails to fully capture the complex interactions between drugs and proteins through simple feature concatenation. Future research should focus on optimizing the GNN architecture and exploring additional suitable feature fusion strategies to more accurately model these intricate interactions. Incorporating substructure information from both drugs and proteins, along with developing a more sophisticated interaction model, is expected to enhance the effectiveness of drug-target interaction (DTI) prediction.

In conclusion, to optimize the utilization of relevant information regarding drugs and proteins and to enhance the predictive efficacy of Drug-Target Interaction (DTI), this study employs feature fusion through multi-modal feature extraction. Specifically, the molecular structure and biological activity characteristics of drugs are extracted using the Mol2vec [24] embedding method and a deep neural network [25]. Mol2vec, based on word embedding techniques, transforms drug molecules into low-dimensional vectors, effectively capturing information about molecular topology and chemical properties. The deep neural network embedding method learns more abstract and complex representations of drug features. For the protein component, we incorporate manually designed amino acid sequence features [26] and sequence embedding features derived from anc2vec [27]. The manually designed features include amino acid composition and secondary structure, which reflect the physical and chemical properties of proteins. Anc2vec maps protein sequences into a low-dimensional vector space, facilitating the identification of similarities and potential functional relationships between sequences.

The integration of diverse modes facilitates a comprehensive understanding of the complex interactions between drugs and proteins, thereby enhancing the accuracy and reliability of drug-target interaction (DTI) predictions. We employ strategies such as stitching and attention mechanisms to merge the extracted features through the relevant feature extraction model. These features are then input into the Multi-Modal Co-Attention (MMCA) model for DTI prediction. An experimental evaluation will follow to assess the effectiveness of this multi-modal feature fusion approach. We aim for this method to significantly improve DTI prediction, providing more precise and reliable support for drug discovery and optimization processes.

## Materials and methods

### Datasets

#### PPIs datasets.

Protein-protein interactions (PPIs) are fundamental to understanding biological processes and are essential for elucidating protein characteristics. Within cellular environments, protein assemblies form through these interactions, significantly influencing biological functions, signal transduction, and metabolism. To clarify the nature and function of these interactions, feature extraction has become a critical endeavor, facilitating the understanding of protein properties and predicting their behavior.

In PPI research, feature extraction involves deriving meaningful information from a protein’s sequence, structure, and interaction network. Relevant characteristics include amino acid composition, secondary structure, spatial conformation, and binding sites with other proteins. By analyzing these features, researchers can gain deeper insights into protein functions and their roles within the cell. Additionally, the emergence of omics technologies, including high-throughput sequencing and protein analysis, has resulted in a substantial increase in data, posing significant challenges to traditional analytical methods. Consequently, the development of efficient feature extraction technologies is of paramount importance.

In recent years, the application of machine learning and deep learning technologies has opened new avenues for feature extraction from protein-protein interactions (PPIs). These algorithms can process large datasets and automatically identify key features influencing protein interactions. For example, the use of convolutional neural networks (CNNs) and recurrent neural networks (RNNs) facilitates the extraction of both local and global features from protein sequences, thereby improving the accuracy of PPI predictions. This approach not only enhances feature extraction efficacy but also serves as a powerful tool for predicting protein functions and identifying novel drug targets. To effectively integrate manually constructed features related to protein sequence and structural information with sequence embedding features based on anc2vec, we utilize the human PPI dataset from the STRING database [28] and employ a transformer model for feature extraction.

The dataset description is as follows:

The human protein-protein interaction (PPI) data is sourced from the STRING database and consists of three columns: protein1, protein2, and combined_score. For additional details, please refer to Table 1.

**Table 1:** Data statistics of PPI dataset.

The number of protein types	The total number of protein-protein relationship pairs	The number of protein-protein pairs with relationships	The number of protein-protein pairs without relationships
18465	13087504	11991230	1096274

To integrate hand-crafted features and sequence embedding features based on anc2vec through PPI training, the task is reformulated as a binary classification problem, specifically predicting whether two proteins interact. A new column, labeled y, is introduced to indicate the presence of an interaction between the two proteins. If the combined_score is less than 500, y is assigned a value of 0, indicating no interaction. Conversely, if the combined_score is 500 or greater, y is assigned a value of 1, indicating an interaction.

#### DDIs datasets.

A drug-drug interaction (DDI) is defined as the interaction between two or more drugs in vivo, potentially impacting their efficacy and safety. Given the increasing number of available pharmaceuticals and the prevalence of polypharmacy, the investigation of DDIs has emerged as a critical area of research. A thorough understanding of DDIs can optimize clinical treatment outcomes while reducing the incidence of adverse reactions, thereby enhancing patient safety. Consequently, extracting effective drug characteristics from DDIs has become an essential component of drug research, development, and clinical application.

In DDI research, feature extraction primarily focuses on the chemical structure, pharmacological properties, and metabolic pathways of drugs within organisms. These characteristics may include molecular fingerprints, binding sites, pharmacokinetic properties, and their interactions with biological targets. By analyzing these features, researchers can identify potential DDIs and assess their impact on patient health. Furthermore, advancements in pharmacogenomics and high-throughput screening technologies continually enhance the capacity to gather extensive drug characteristic data, providing a valuable resource for DDI research.

The emergence of machine learning and deep learning technologies has created new opportunities for extracting features related to drug-drug interactions (DDIs). These algorithms efficiently process complex drug datasets and automatically identify key features that influence DDIs. For instance, the application of a graph neural network (GNN) and a deep learning model facilitates the extraction of intricate features from the molecular structures of drugs, thereby improving the accuracy of DDI predictions. This approach not only accelerates feature extraction but also provides valuable insights for the design of new drugs and the repurposing of existing ones.

In our study on drug feature extraction, we utilize the drug-drug interaction dataset compiled by Bacciu et al. and the CLAIRE Alliance [25]. We employ a graph convolutional network (GCN) model to train and learn drug embedding features derived from mol2vec and a deep neural network, with the aim of obtaining the final fused feature results. A comprehensive description of this dataset is provided in Table 2.

**Table 2:** Data statistics of DDI dataset.

The number of drug types	The total number of drug-drug relationship pairs	The number of drug-drug pairs with relationships	The number of drug-drug pairs without relationships
616	11940	604	11336

#### DTIs datasets.

Drug-target interaction (DTI) refers to the relationship between drug molecules and their biological targets, typically proteins or other biological entities. This field is crucial for drug discovery and development, especially in drug repositioning, or “drug reuse,” which involves applying approved drugs to new indications or targets. By predicting interactions between drugs and targets, researchers can identify potential new uses, thereby accelerating drug development and reducing costs.

The primary objective of DTI research is to extract features, including the chemical structure of drugs, the amino acid sequences of targets, the characteristics of binding sites, and the pharmacological properties of drugs. Effective extraction and analysis of these features facilitate the creation of models that correlate drugs with their targets, enabling the prediction of new drug-target interactions. Additionally, researchers can utilize bioinformatics tools and databases to integrate extensive data on drugs and targets, enhancing the accuracy and efficiency of the analytical process.

In recent years, advancements in computing power and the evolution of data science have significantly enhanced the application of machine learning and deep learning methodologies in drug-target interaction (DTI) research. These methodologies are adept at managing complex, multidimensional datasets and can automatically identify key features influencing drug-target interactions. For instance, developing a prediction model based on drug structures and target properties allows for the rapid identification of drugs with potential repositioning value. This progress has notably expedited the drug repositioning process and provided essential insights for the development of novel pharmaceuticals.

In this study, we utilize the BioSNAP [29], BindingDB [30], and the human DTI dataset from the STRING database [27] to evaluate the effectiveness of our feature fusion strategy and the MMCA model. A detailed overview of the datasets is provided in Tables 3, 4 and 5.

**Table 3:** Data statistics of BioSNAP dataset.

The number of drug types	The number of protein types	The total number of drug-protein relationship pairs	The number of drug-drug pairs with relationships
4526	2101	13856	13856

**Table 4:** Data statistics of BindingDB dataset.

The number of drug types	The number of protein types	The total number of drug-protein relationship pairs	The number of drug-drug pairs with relationships
303	498	19932	19932

**Table 5:** Data statistics of Human dataset.

The number of drug types	The number of protein types	The total number of drug-protein relationship pairs	The number of drug-drug pairs with relationships
750	2041	13910	13910

### Performance evaluation metrics

To conduct a thorough evaluation of our model and strategy across various data sets, we employ several assessment indicators, including accuracy, sensitivity, specificity, precision, recall, F1 score, AUC, and AUPR. To ensure the most appropriate evaluation, we select indicators based on their characteristics and the specific requirements of the analysis. This targeted evaluation method facilitates a deeper understanding of the model’s performance in different scenarios, providing a solid foundation for further optimization and improvement.

## Overall framework of model

To conduct a thorough evaluation of our model and strategy across various data sets, we employ several assessment indicators, including accuracy, sensitivity, specificity, precision, recall, F1 score, AUC, and AUPR. To ensure the most appropriate evaluation, we select indicators based on their characteristics and the specific requirements of the analysis. This targeted evaluation method facilitates a deeper understanding of the model’s performance in different scenarios, providing a solid foundation for further optimization and improvement.

### Protein feature extraction

In constructing protein features, we utilize both the manually designed amino acid sequence features and the sequence embedding features derived from Anc2Vec.

#### Anc2vec feature construction.

Initially, gene expressions associated with the Gene Ontology (GO) terms for all human proteins are downloaded from the STRING database. These expressions are then mapped to the protein features in the protein-protein interaction (PPI) dataset, establishing a correlation between the two. Subsequently, the final form of the protein GO gene expression is extracted by constructing the feature using Anc2vec. The Anc2vec is constructed as follows:

Let $X\in {\left\{0,1\right\}}^{n_{x}}$ represent the one-hot vector of the input term. The input *x* is transformed into the embedding vector $W_{x}=h\in R^{n_{h}}$ using the weight matrix $W\in R^{n_{h}\cdot n_{x}}$. By defining $n_{h}\ll n_{x}$, the resulting embedding is low-dimensional, significantly reducing the size of *W*. The vector *h* is then utilized to construct three vectors for weight optimization, as described by the following formulas:


$$\begin{array}{c} {\hat{y}}^{r}=\phi (Rh+r) \end{array}$$ (1)

$$\begin{array}{c} {\hat{y}}^{s}=\phi (Sh+s) \end{array}$$ (2)

$$\begin{array}{c} {\hat{y}}^{a}=\phi (Ah+a) \end{array}$$ (3)

Here, $R\in R^{n_{h}\dot{n_{x}}},S\in R^{3\dot{n_{x}}}$ and $A\in R^{n_{h}\dot{n_{x}}}$ are the additional weight matrices, with *r*, *s*, and *a* representing the corresponding bias vectors. The softmax function ϕ(·) provides the probability distribution of the vector representation.

#### Handcrafted sequence characteristics.

We also downloaded the sequence information for all human proteins from the STRING database, mapped it to the PPI dataset, and encoded it using manually designed amino acid sequence features to create the final feature representation. This feature coding includes the construction of APAACplus, which utilizes the tripeptide sequence. The formula is as follows:


$$\begin{array}{c} A=[\frac{f_{1}}{C},...,\frac{f_{20}}{C},\frac{w_{1}\tau_{21}}{C},...,\frac{w_{1}\tau_{20+2\lambda }}{C},\frac{w_{2}\tau_{21+2\lambda }}{C},...,\frac{w_{2}\tau_{21+4\lambda }}{C}] \end{array}$$ (4)

where $C=𝛴f_{i}+w1𝛴\tau_{d}+w2𝛴v_{d}$; $f_{r}$ fr represents the normalized frequency of the 20 amino acids in the input protein; $\tau_{d}$ and $v_{d}$ indicate the sequence correlation between all dipeptides and tripeptides in the input protein, respectively; and *w*_1_ and *w*_2_ are weighting factors set at 0.5 each for this study. Finally, we employed the PPI dataset and its transformer model for training, using the two types of protein feature information we constructed as input to the model, from which we extracted the trained final feature as the protein input feature for the DTI task training.

### Drug feature extraction

We utilize the mol2vec embedding method and deep neural networks to extract the molecular structure and biological activity characteristics of drugs.

#### Feature embedding of deep neural network.

Bacciu et al. [25] assert that the properties of drugs primarily encompass information regarding atoms and bonds. For atoms, they utilize one-hot encoding for the symbol, hybridization type, hydrogen count, and bonding degree. For bonds, they consider one-hot encoding for stereochemistry, bond type (single, double, triple, or aromatic), and whether the bonds are part of a ring. To effectively capture the structural and topological characteristics of drugs, we employ a three-layer Drug Graph Network (DGN), referred to as a Prediction Network based on Structural Similarity (SSN). Both drug embedding modules produce 96-dimensional embedding vectors to comprehensively represent the characteristics of the drugs.

#### Mol2vec characteristic construction.

Mol2Vec is a pre-trained molecular encoder that utilizes a concept similar to word embedding to learn the substructures of various compounds, thereby generating molecular representations. It converts the SMILES (Simplified Molecular Input Line Entry Specification) notation of a compound into a corresponding 300-dimensional vector encoded by Mol2Vec. This encoding method employs key technologies from Word2Vec, including hierarchical softmax, which organizes words based on frequency using a Huffman tree. In this approach, high-frequency words are positioned closer to the root node, while low-frequency words are placed further away. Consequently, during training, the parameters along the paths of low-frequency words receive more attention, enhancing the model’s generalization capabilities. As a result, Mol2Vec effectively captures essential information within molecular structures, providing robust support for subsequent chemical analysis and predictive tasks.

Similar to word2vec, which learns vector representations of words in text, Mol2vec is a molecular embedding method that learns low-dimensional vector representations of molecular substructures. The process of Mol2vec involves the following steps:

- Define a Molecular Vocabulary: Mol2vec begins by establishing a vocabulary of molecular substructures, akin to a vocabulary of words in a text. This vocabulary encompasses atoms, bonds, rings, and other molecular fragments.
- Generate Molecular “Sentences”: Each molecule is represented as a “sentence” composed of these molecular substructures, where the sequence of substructures reflects the connectivity and topological structure of the molecule.
- Train the Mol2vec Model: The next step involves training the Mol2vec model to learn the vector representation of each molecular substructure, thereby optimizing predictions of neighboring substructures within a given molecule. Upon completion of training, each molecule can be represented by a low-dimensional vector that encapsulates its structural and topological characteristics.

The Mol2vec formula is expressed as follows:


$$\begin{array}{c} 𝐯mol=f(m)=\frac{1}{\left|m\right|}\sum {i=1}^{\left|m\right|}{𝐯}_{frag_{i}} \end{array}$$ (5)

Where 𝐯mol denotes the vector representation of molecule *m*, and $𝐯frag_{i}$ represents the vector of the *i*-th substructure fragment in molecule *m*.

### MMCA model and overall architecture

#### MMCA model.

To enhance the efficacy of Drug-Target Interaction (DTI) prediction, we introduce an innovative Multi-Modal Co-Attention (MMCA) model. This model employs a comprehensive approach to utilize diverse information related to drugs and proteins through meticulous mining and optimization. This process facilitates subsequent feature fusion and model training. Specifically, the MMCA model initially extracts and processes the original drug and protein features separately.

For drug feature extraction, the SG_GNN sub-module employs the SAGEConv [31] and GATConv [32] modules of the graph neural network, enabling the effective extraction of complex hidden features from the drug’s molecular structure. In terms of protein features, the LTDTIModel submodule, based on a linear encoder, self-attention mechanism, and linear decoder, optimizes and enriches the representation of protein sequence features.

In contrast to simple fusion strategies such as feature concatenation or element-wise operations, which treat drug and protein features as independent and static vectors, our co-attention mechanism dynamically aligns and weights cross-modal dependencies. This design allows the model to focus on biologically relevant substructures and interaction motifs that govern drug-target binding, rather than combining features in a fixed manner. Such adaptive modeling better captures the complex, context-dependent relationships between drugs and proteins, leading to more accurate and robust DTI prediction.

By utilizing these targeted feature extraction and optimization techniques, the MMCA model fully exploits the multi-faceted information inherent to both drugs and proteins. The two optimized features are subsequently fused through concatenation, and their interaction is predicted using an MLP decoder.

**Fig 1:**
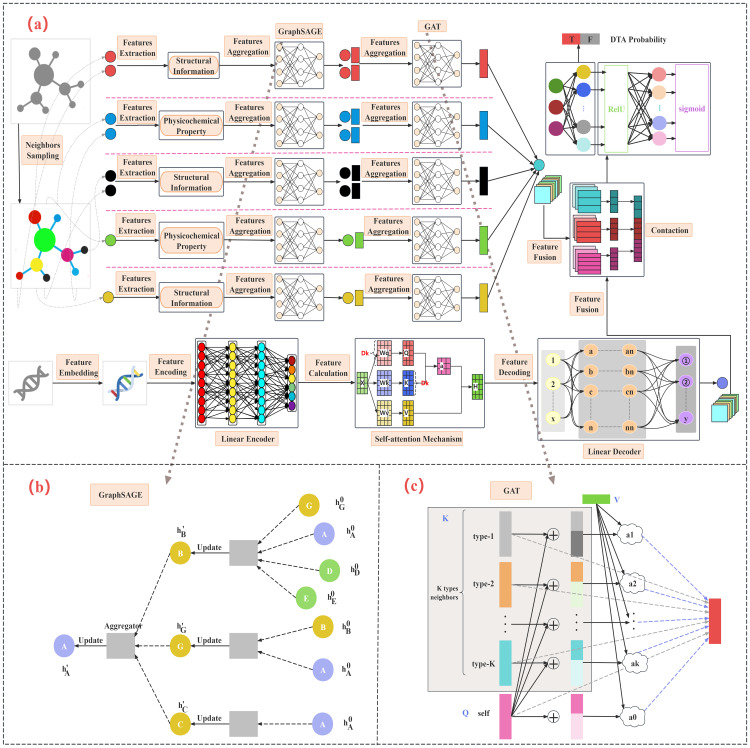
Architecture of the MMCA model’s dual-path encoding and fusion pipeline. The framework consists of two parallel branches: (a) a graph-based drug encoder that leverages GAT and GraphSAGE to extract topological and chemical features from molecular graphs, and (b) a protein sequence encoder that integrates handcrafted physicochemical descriptors with learned embeddings. Each branch performs hierarchical feature extraction through multiple layers of neighborhood aggregation and nonlinear transformation. The resulting modality-specific representations are then fused via a cross-modal interaction module (highlighted in the center), which enables contextual alignment and joint refinement before final prediction.

The flow chart of the MMCA model is presented in Fig 1.

#### Overall architecture.

The study presents a comprehensive framework for predicting drug-target relationships, consisting of three primary components:

(1) Protein Feature Extraction Using a Transformer Model: We utilize the Transformer network to extensively train protein features derived from anc2vec and manual feature engineering techniques. This approach aims to extract more meaningful representations from protein sequence and structural information, establishing a robust foundation for the subsequent drug-target interaction (DTI) prediction task.
(2) Optimization of Drug Features Using a Graph Convolutional Network (GCN): We implement the GCN model to enhance drug features initially constructed through deep learning and mol2vec. This process involves a thorough examination of the structural topology, electronic properties, physicochemical characteristics, and other relevant aspects of drug molecules. The extraction of rich drug feature representations significantly supports the subsequent DTI analysis and prediction tasks.
(3) The DTI predicted a fusion of features: The final extracted protein and drug features are combined and input into the MMCA model for comprehensive drug-target interaction prediction. This framework leverages the full potential of deep learning to process structured biomolecular data, effectively integrating multi-source information on proteins and drugs, thereby offering invaluable support for applications such as new drug discovery.

**Fig 2:**
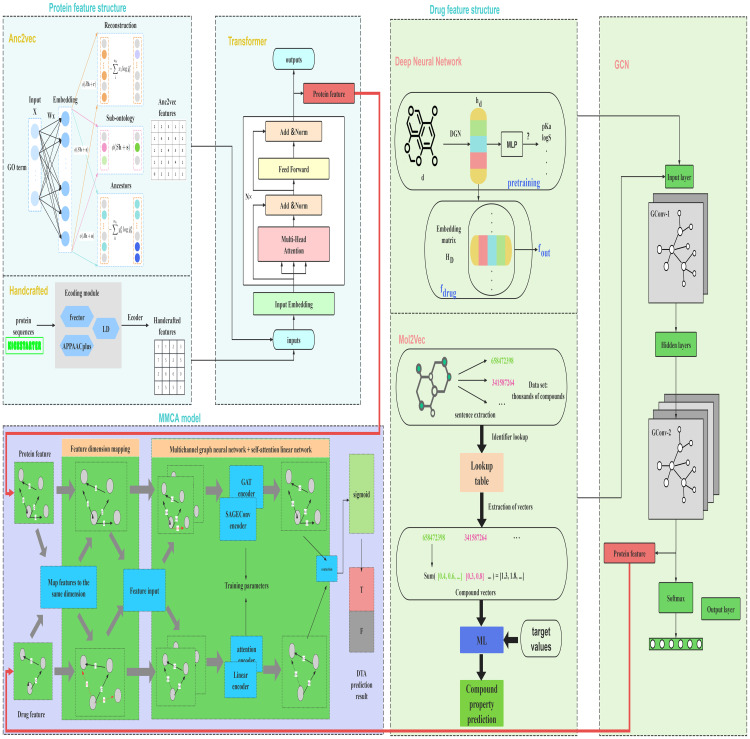
Overall framework of the MMCA model for drug–target interaction prediction. The model takes raw drug SMILES and protein sequences as input. Drug molecules are converted into graphs and processed by a GNN stack (including GCN and GAT layers) to obtain structural embeddings; proteins are encoded through a hybrid strategy combining sequence-derived vectors and engineered features. These heterogeneous representations are projected into a shared latent space, where a multi-layer perceptron (MLP) with attention-guided fusion computes the interaction probability. The entire architecture is trained end-to-end to optimize binary DTI classification, enabling unified learning of cross-modal dependencies.

The overall framework is illustrated in Fig 2.

## Experimental results and discussion

### PPI feature extraction

We extract protein features from the STRING database. Subsequently, we employ a transformer model to further extract and represent these features, confirming that the constructed features perform well on the dataset. To better illustrate the advantages of the feature fusion, we conduct ablation experiments, with the results presented in Table 6.

**Table 6:** PPI ablation experiment.

Model	Description	Accuracy	Sensitivity	Specificity	Recall	AUC
Transformer(ours)	Anc2vec + handcrafted sequence feature	**93.91**% ±**0.02**%	99.28% ±0.02%	**93.42**% ±**0.02**%	99.28% ±0.02%	**96.35**% ±**0.01**%
Transformer-1	Only anc2vec feature	92.88% ±0.01%	**99.30**% ±**0.01**%	92.30% ±0.01%	**99.30**% ±**0.01**%	95.80% ±0.01%
Transformer-2	Only handcrafted sequence feature	89.18% ±0.02%	99.05% ±0.01%	88.27% ±0.02%	99.05% ±0.01%	93.66% ±0.01%

The results of the ablation experiments indicate that combining anc2vec features with hand-compiled sequence features produces optimal outcomes, outperforming models that utilize a single feature. This is evidenced by improved accuracy, sensitivity, specificity, recall, and AUC values. Such a combination effectively captures the complex patterns of internal information within proteins, thus enhancing predictive performance. In contrast, the Transformer-1 model, which relies solely on anc2vec features, demonstrates strong sensitivity and recall but exhibits relatively lower accuracy, specificity, and AUC values. This suggests that while anc2vec features can effectively convey structural and functional information about proteins, they do not fully encompass the entire protein interaction landscape. On the other hand, the Transformer-2 model, which relies exclusively on manual sequence features, shows the poorest performance across all metrics. This indicates that the nuances of protein interactions cannot be adequately captured by manual features alone. Although these features may intuitively reflect certain structural characteristics, they struggle to represent the underlying implicit principles governing protein interactions. Consequently, feature fusion serves as an effective strategy that fully leverages the strengths of various features, thereby improving the accuracy of protein interaction predictions.

### DDI feature extraction

We extract features from the drug-drug interaction dataset compiled by Bacciu et al. and the CLAIRE Alliance. We then use the Graph Convolutional Network (GCN) model to optimize and integrate these features, confirming that the final extracted features exhibit strong performance on this dataset. To illustrate the advantages and disadvantages of various feature fusion methods, we conduct ablation experiments, with the results presented in Table 7.

**Table 7:** DDI ablation experiment.

Model	Description	Accuracy	Sensitivity	Specificity	Precision	Recall	F1-score	AUC	AUPR
GCN(ours)	Feature embedding + Mol2vec feature	**91.37**% ±**1.50**%	**92.55**% ±**4.22**%	**90.29**% ±**4.44**%	**90.67**% ±**3.88**%	**92.55**% ±**4.22**%	**91.44**% ±**1.51**%	**97.13**% ±**0.58**%	**96.79**% ±**0.70**%
GCN-1	Only feature embedding	88.85% ±1.94%	89.03% ±2.42%	88.67% ±3.52%	88.78% ±3.17%	89.03% ±2.42%	88.85% ±1.86%	95.86% ±0.98%	95.88% ±1.03%
GCN-2	Only mol2vec feature	87.14% ±0.26%	86.11% ±3.35%	88.23% ±2.95%	88.01% ±2.44%	86.11% ±3.35%	86.95% ±0.50%	94.80% ±0.39%	94.79% ±0.65%

The results of the ablation experiments indicate that feature fusion is essential for enhancing the potential characteristics of drugs. The GCN model exhibits outstanding performance across various metrics, particularly in accuracy, sensitivity, and AUC value, due to the integration of feature embedding and Mol2vec features. This suggests that combining different types of features effectively captures the intricate internal patterns and associations in drug interaction data. In contrast, models that rely on a single feature, such as GCN-1 and GCN-2, yield favorable outcomes; however, certain indicators, including specificity and accuracy, remain inadequate. This underscores that a single feature is insufficient for fully capturing the complexities of drug interactions, highlighting the necessity for multiple feature fusion to accurately represent this complex biological process. The advantage of feature fusion lies in its ability to leverage the strengths of various features, compensating for the limitations of individual ones. In the context of the DDI task, the use of feature embedding facilitates the capture of information related to the structural and physicochemical properties of drug molecules. Conversely, Mol2vec features represent the characteristics of drug interactions based on the chemical structures of the relevant compounds. The integration of these two distinct features enhances the GCN model’s ability to predict drug-drug interactions (DDIs) more comprehensively and accurately.

### DTI prediction task

We utilize the final extracted protein and drug features as input for the MMCA model, verifying the model on the BioSNAP, BindingDB, and human DTI datasets obtained from the STRING database. We conduct both comparative and ablation experiments on the MMCA model. In the comparative experiments, we select several popular models relevant to the characteristics of the different datasets for comparison. The results of these comparative experiments are presented in Tables 8, 9 and 10, while the conclusions from the ablation experiments are summarized in Table 11.

**Table 8:** Comparative experiment of DTI task on BioSNAP dataset.

Model	Accuracy	Sensitivity	Specificity	F1-score	AUC
SVM [33]	75.0% ±0.050%	66.5% ±0.046%	83.5% ±0.054%	82.7% ±0.053%	81.9% ±0.045%
RF [34]	79.3% ±0.001%	76.3% ±0.002%	83.8% ±0.011%	78.7% ±0.001%	85.7% ±0.001%
Graph-DTA [23]	80.0% ±0.005%	76.1% ±0.015%	83.8% ±0.011%	80.7% ±0.005%	87.1% ±0.001%
Transformer-CPI [35]	79.7% ±0.008%	76.8% ±0.024%	82.7% ±0.012%	80.3% ±0.006%	87.6% ±0.004%
MolTrans [36]	82.0% ±0.011%	79.1% ±0.032%	84.8% ±0.014%	82.5% ±0.007%	89.5% ±0.006%
MMCA (ours)	**90.14**% ±**0.39**%	**89.52**% ±**0.96**%	**90.76**% ±**1.26**%	**90.08**% ±**0.36**%	**90.14**% ±**0.39**%

**Table 9:** Comparative experiment of DTI task on BindingDB dataset.

Model	Accuracy	Sensitivity	Specificity	F1-score	AUC	AUPR
SVM [33]	82.4% ±0.001%	77.6% ±0.000%	85.7% ±0.002%	78.5% ±0.000%	90.4% ±0.000%	86.5% ±0.001%
RF [34]	87.1% ±0.001%	84.0% ±0.002%	89.3% ±0.002%	84.4% ±0.002%	94.2% ±0.001%	92.3% ±0.001%
GraphDTA [23]	87.4% ±0.010%	85.8% ±0.026%	89.7% ±0.014%	88.0% ±0.005%	94.4% ±0.004%	92.3% ±0.006%
TransformerCPI [35]	88.8% ±0.007%	88.6% ±0.016%	89.0% ±0.008%	88.8% ±0.005%	94.7% ±0.004%	93.2% ±0.004%
MolTrans [36]	88.4% ±0.007%	87.7% ±0.018%	89.4% ±0.014%	88.6% ±0.005%	94.7% ±0.004%	92.7% ±0.006%
DrugBAN [37]	90.1% ±0.003%	89.4% ±0.011%	90.8% ±0.009%	90.3% ±0.001%	96.1% ±0.001%	94.8% ±0.001%
CAT-DTI [38]	89.6% ±0.002%	88.4% ±0.010%	91.3% ±0.009%	90.0% ±0.001%	96.0% ±0.001%	94.7% ±0.001%
DeepCDA [39]	83.1% ±0.011%	87.2% ±0.013%	84.0% ±0.004%	81.1% ±0.005%	89.4% ±0.003%	90.1% ±0.012%
TripletMultiDTI [40]	86.5% ±0.005%	91.7% ±0.022%	86.3% ±0.001%	84.0% ±0.002%	93.1% ±0.001%	94.0% ±0.003%
GAT [32,41]	77.5% ±0.012%	88.7% ±0.001%	85.1% ±0.001%	75.5% ±0.021%	91.3% ±0.001%	92.3% ±0.002%
MMCA (ours)	**98.37**% ±**0.33**%	**99.31**% ±**0.21**%	**97.43**% ±**0.75**%	**98.39**% ±**0.32**%	**98.37**% ±**0.33**%	**97.15**% ±**0.66**%

**Table 10:** Comparative experiment of DTI task on Human dataset.

Model	Accuracy	Sensitivity	Specificity	F1-score
SVM [33]	83.8% ±0.000%	78.2% ±0.000%	83.0% ±0.000%	81.1% ±0.000%
RF [34]	86.6% ±0.006%	83.3% ±0.006%	89.3% ±0.007%	84.8% ±0.005%
GraphDTA [23]	90.8% ±0.008%	91.2% ±0.017%	90.4% ±0.016%	90.7% ±0.008%
Transformer-CPI [35]	87.9% ±0.007%	83.1% ±0.023%	93.9% ±0.018%	89.1% ±0.005%
MolTrans [36]	94.01% ±0.004%	94.9% ±0.011%	93.9% ±0.017%	94.3% ±0.005%
DrugBAN [37]	94.0% ±0.003%	93.8% ±0.010%	94.1% ±0.013%	94.0% ±0.004%
CAT-DTI [38]	94.2% ±0.002%	92.9% ±0.007%	**95.7**% ±**0.008**%	94.4% ±0.002%
MMCA (ours)	**94.61**% ±**0.43**%	**95.36**% ±**0.77**%	93.86% ±0.84%	**94.65**% ±**0.43**%

**Table 11:** Ablation experiment of DTI task on BindingDB dataset.

Model	Describe	Accuracy	Sensitivity	Specificity	F1-score	AUC	AUPR
MMCA-1	Only self-attention linear networks	92.91% ±0.19%	98.22% ±0.07%	87.61% ±0.39%	93.26% ±0.17%	92.92% ±0.19%	88.09% ±0.30%
MMCA-2	Only Graph neural network	91.20% ±2.70%	84.69% ±5.51%	**97.72**% ±**0.15**%	90.49% ±3.29%	91.20% ±2.70%	90.13% ±2.64%
MMCA-3	Point multiplication classifier	97.93% ±0.31%	98.18% ±0.33%	97.67% ±0.51%	97.93% ±0.31%	97.93% ±0.31%	96.82% ±0.52%
MMCA (ours)	GNN + self-attention linear networks + MLP Classifier	**98.37**% ±**0.33**%	**99.31**% ±**0.21**%	97.43% ±0.75%	**98.39**% ±**0.32**%	**98.37**% ±**0.33**%	**97.15**% ±**0.66**%

All experiments are conducted with fixed experimental configurations to ensure consistency and reproducibility. Specifically, the batch size is set to 3840, the learning rate is $1\times 10^{-3}$, and the Adam optimizer is applied with a weight decay of $1\times 10^{-5}$. The model is trained for up to 300 epochs with a hidden dimension of 64 and two GNN layers. The number of sampled neighbors is set to 20 and 10, the random seed is fixed at 50, and dropout rates of 0.2 and 0.6 are used in different network components. All implementations are based on Python 3.9 and PyTorch 1.13 + , and run on a GPU environment.

#### Comparative experiment.

A comprehensive analysis of the experimental results across three datasets reveals that the MMCA model excels in all performance indicators, highlighting its exceptional capabilities in drug-target interaction (DTI) forecasting tasks. The results display that the MMCA model outperforms other models, including SVM, RF, GraphDTA, Transformer-CPT, and MolTrans, particularly on the BioSNAP datasets. This underscores the model’s ability to accurately and comprehensively capture the complex patterns of drug-target interactions, providing an efficient and reliable solution for DTI prediction tasks. In contrast, while other models exhibit satisfactory performance, they do not match the overall effectiveness of the MMCA model. In the BindingDB dataset, the MMCA model achieves accuracy, sensitivity, specificity, and other key metrics exceeding 90%. It also excels in the F1 score, AUC, and AUPR, demonstrating its capability to accurately identify interactions between drugs and targets, making it a reliable tool for clinical applications. The MMCA model performs exceptionally well on the Human dataset, with accuracy, sensitivity, specificity, and F1 score all surpassing 93%. It outperforms other models in several metrics, further confirming the stability and generalizability of the MMCA model across different datasets. Although individual indicators like DrugBAN and CAT-DTI also show significant sensitivity and specificity, the MMCA model remains the most effective across all three datasets.

#### Ablation experiment.

We present a detailed comparison of the performance of various variants of the MMCA model using the BindingDB dataset. The results demonstrate that the MMCA model proposed in this study is the most effective overall, achieving the highest levels of accuracy, sensitivity, specificity, F1 score, AUC, and AUPR. Although there is a slight decline in the specificity index, this does not diminish the model’s overall performance. Furthermore, the model’s exceptional ability to balance the recognition of interactive and non-interactive samples underscores its potential utility in the field of drug-target interaction (DTI) analysis. This evidence conclusively demonstrates that the MMCA model is capable of accurately and comprehensively capturing the intricate patterns of drug-target interactions, thereby offering an efficient and reliable solution for DTI prediction tasks.

## Case study

To evaluate the model’s reliability, we selected a subset of drugs and proteins from the BioSNAP dataset and analyzed its drug–target interaction (DTI) prediction results. Specifically, we randomly chose two drugs—ropinirole and tretinoin—each with at least ten known targets, and examined the model’s predictive accuracy for them. As shown in Table 12, which lists the model-predicted targets, actual interaction labels, and prediction results, the model achieved 100% prediction accuracy for both drugs. All predictions perfectly matched the true labels, with no mispredictions occurring.

**Table 12:** Prediction results for the drugs Ropinirole and Tretinoin.

Drug	Protein	Actual label	Predicted label
DB00268-Ropinirole	P41231	False	False
	P08913	True	True
	P41595	True	True
	P21917	True	True
	Q9UQL6	False	False
	P28222	True	True
	P10635	True	True
	P05177	True	True
	P18089	True	True
	P35462	True	True
	P21728	True	True
DB00755-Tretinoin	P48443	True	True
	P13631	True	True
	P00352	True	True
	P11712	True	True
	P04798	True	True
	P08684	True	True
	P24462	True	True
	P33260	True	True
	P10632	True	True
	P28702	True	True

## Conclusion

This study introduces the MMCA model for predicting drug-target interactions (DTIs), which significantly improves the accuracy and robustness of DTI forecasting by integrating multi-modal features such as drug molecular structure and protein sequence information. Traditional DTI prediction methods often struggle to effectively integrate heterogeneous data sources. MMCA overcomes this challenge by leveraging a multi-modal co-attention mechanism to dynamically align and combine drug and protein features, enabling the model to capture both structural and semantic cues in parallel.

Our experimental results on three widely used benchmark datasets — BioSNAP, BindingDB and Human STRING — demonstrate that MMCA outperforms existing state-of-the-art methods. It achieves superior performance in multiple evaluation metrics, including AUC, AUPR and F1-score. These results highlight the model’s ability to accurately model complex biological relationships and predict drug-target interactions. Notably, MMCA achieved an AUC of up to 98.4% on the BindingDB dataset, demonstrating its strong generalisability across different datasets and biological domains.

Ablation studies reveal the critical role of the co-attention fusion module in improving model performance. Specifically, combining drug features derived from graph-based representations with protein features obtained from sequence embeddings provides a more comprehensive understanding of drug-target interactions. The model’s ability to adaptively weight cross-modal dependencies further improves its robustness, enabling it to make reliable predictions even when data is noisy or incomplete.

We further quantify the relative contributions of multimodal feature design and the co-attention mechanism. Results show that multimodal feature extraction provides the foundational performance gain by enriching representations of drugs and proteins, while the co-attention mechanism delivers an additional boost by dynamically aligning cross-modal interactions. Both components are indispensable: multimodal features supply comprehensive information, and co-attention effectively models their complex relationships. Together, they synergistically enhance prediction accuracy, with neither component alone achieving the full performance of the complete MMCA model.

One of MMCA’s key advantages is its flexibility in handling diverse types of biological data, making it ideal for broader applications in drug discovery, drug repositioning and other bioinformatics tasks. The biologically plausible interactions identified by MMCA in its high-confidence predictions suggest its potential to guide experimental validation efforts and thereby accelerate the drug discovery process. Furthermore, the model’s architecture facilitates end-to-end multimodal reasoning and provides a scalable, adaptable framework for future advancements in DTI prediction and related domains.

To support practical deployment, we further analyzed the computational efficiency of MMCA. On a standard GPU environment, the model completes training within 10 hours on the largest dataset, with inference times of less than 0.1 seconds per drug-target pair, making it suitable for batch screening in early-stage drug discovery. However, scaling to extremely large datasets or deploying in low-resource clinical settings remains challenging due to memory footprint and inference latency constraints. These practical considerations highlight the need for further optimization, such as lightweight model pruning and knowledge distillation, to balance predictive performance with computational cost.

However, our approach has certain limitations. Reliance on large-scale annotated datasets for training and evaluation remains challenging, as obtaining such datasets is resource-intensive. Future work could explore integrating additional biological data sources, such as gene expression profiles or omics data, to enhance MMCA’s predictive capabilities further. While the co-attention mechanism significantly improves prediction performance, the interpretability of the model’s decisions could be enhanced further through advanced visualisation techniques or the development of explainable AI methods.

## References

[1] Agamah FE, Mazandu GK, Hassan R, Bope CD, Thomford NE, Ghansah A, et al. Computational/in silico methods in drug target and lead prediction. Brief Bioinform. 2020;21(5):1663–75. doi: 10.1093/bib/bbz103 31711157 PMC7673338

[2] Zhao Q, Yang M, Cheng Z, Li Y, Wang J. Biomedical Data and Deep Learning Computational Models for Predicting Compound-Protein Relations. IEEE/ACM Trans Comput Biol Bioinform. 2022;19(4):2092–110. doi: 10.1109/TCBB.2021.3069040 33769935

[3] Pan X, Lin X, Cao D, Zeng X, Yu PS, He L, et al. Deep learning for drug repurposing: Methods, databases, and applications. WIREs Comput Mol Sci. 2022;12(4):e1597. doi: 10.1002/wcms.1597

[4] Overington JP, Al-Lazikani B, Hopkins AL. How many drug targets are there? Nature reviews Drug discovery. 2006;5(12):993–6. 17139284 10.1038/nrd2199

[5] Hu S, Zhang C, Chen P, Gu P, Zhang J, Wang B. Predicting drug-target interactions from drug structure and protein sequence using novel convolutional neural networks. BMC Bioinformatics. 2019;20(Suppl 25):689. doi: 10.1186/s12859-019-3263-x 31874614 PMC6929541

[6] Kramer C, Kalliokoski T, Gedeck P, Vulpetti A. The experimental uncertainty of heterogeneous public K(i) data. J Med Chem. 2012;55(11):5165–73. doi: 10.1021/jm300131x 22643060

[7] Friesner RA, Banks JL, Murphy RB, Halgren TA, Klicic JJ, Mainz DT, et al. Glide: a new approach for rapid, accurate docking and scoring. 1. Method and assessment of docking accuracy. J Med Chem. 2004;47(7):1739–49. doi: 10.1021/jm0306430 15027865

[8] Wallach I, Dzamba M, Heifets A. AtomNet: a deep convolutional neural network for bioactivity prediction in structure-based drug discovery. arXiv preprint arXiv:151002855. 2015.

[9] Chen R, Liu X, Jin S, Lin J, Liu J. Machine Learning for Drug-Target Interaction Prediction. Molecules. 2018;23(9):2208. doi: 10.3390/molecules23092208 30200333 PMC6225477

[10] Li W, Ma W, Yang M, Tang X. MFCM-DTI model of multimodal feature fusion: prediction of drug-target interaction. In: 2024 IEEE International Conference on Bioinformatics and Biomedicine (BIBM). IEEE; 2024. p. 687–692.

[11] Himmat M, Salim N, Al-Dabbagh MM, Saeed F, Ahmed A. Adapting Document Similarity Measures for Ligand-Based Virtual Screening. Molecules. 2016;21(4):476. doi: 10.3390/molecules21040476 27089312 PMC6274479

[12] Sieg J, Flachsenberg F, Rarey M. In Need of Bias Control: Evaluating Chemical Data for Machine Learning in Structure-Based Virtual Screening. J Chem Inf Model. 2019;59(3):947–61. doi: 10.1021/acs.jcim.8b00712 30835112

[13] Maia EHB, Assis LC, de Oliveira TA, da Silva AM, Taranto AG. Structure-Based Virtual Screening: From Classical to Artificial Intelligence. Front Chem. 2020;8:343. doi: 10.3389/fchem.2020.00343 32411671 PMC7200080

[14] Su M, Yang Q, Du Y, Feng G, Liu Z, Li Y, et al. Comparative Assessment of Scoring Functions: The CASF-2016 Update. J Chem Inf Model. 2019;59(2):895–913. doi: 10.1021/acs.jcim.8b00545 30481020

[15] Tian K, Shao M, Wang Y, Guan J, Zhou S. Boosting compound-protein interaction prediction by deep learning. Methods. 2016;110:64–72. 27378654 10.1016/j.ymeth.2016.06.024

[16] Lee I, Keum J, Nam H. DeepConv-DTI: Prediction of drug-target interactions via deep learning with convolution on protein sequences. PLoS Comput Biol. 2019;15(6):e1007129. doi: 10.1371/journal.pcbi.1007129 31199797 PMC6594651

[17] Rogers D, Hahn M. Extended-connectivity fingerprints. J Chem Inf Model. 2010;50(5):742–54. doi: 10.1021/ci100050t 20426451

[18] Zhang S, Jiang M, Wang S, Wang X, Wei Z, Li Z. SAG-DTA: Prediction of Drug-Target Affinity Using Self-Attention Graph Network. Int J Mol Sci. 2021;22(16):8993. doi: 10.3390/ijms22168993 34445696 PMC8396496

[19] Zheng S, Li Y, Chen S, Xu J, Yang Y. Predicting drug–protein interaction using quasi-visual question answering system. Nat Mach Intell. 2020;2(2):134–40. doi: 10.1038/s42256-020-0152-y

[20] Wei L, Zou Q, Liao M, Lu H, Zhao Y. A novel machine learning method for cytokine-receptor interaction prediction. Comb Chem High Throughput Screen. 2016;19(2):144–52. doi: 10.2174/1386207319666151110122621 26552440

[21] Wei L, Bowen Z, Zhiyong C, Gao X, Liao M. Exploring local discriminative information from evolutionary profiles for cytokine–receptor interaction prediction. Neurocomputing. 2016;217:37–45. doi: 10.1016/j.neucom.2016.02.078

[22] Wei L, Long W, Wei L. MDL-CPI: Multi-view deep learning model for compound-protein interaction prediction. Methods. 2022;204:418–27. doi: 10.1016/j.ymeth.2022.01.008 35114401

[23] Nguyen T, Le H, Quinn TP, Nguyen T, Le TD, Venkatesh S. GraphDTA: predicting drug-target binding affinity with graph neural networks. Bioinformatics. 2021;37(8):1140–7. doi: 10.1093/bioinformatics/btaa921 33119053

[24] Jaeger S, Fulle S, Turk S. Mol2vec: Unsupervised Machine Learning Approach with Chemical Intuition. J Chem Inf Model. 2018;58(1):27–35. doi: 10.1021/acs.jcim.7b00616 29268609

[25] Bacciu D, Errica F, Gravina A, Madeddu L, Podda M, Stilo G. Deep Graph Networks for Drug Repurposing With Multi-Protein Targets. IEEE Trans Emerg Topics Comput. 2024;12(1):177–89. doi: 10.1109/tetc.2023.3238963

[26] Tran H-N, Nguyen P-X-Q, Guo F, Wang J. Prediction of Protein-Protein Interactions Based on Integrating Deep Learning and Feature Fusion. Int J Mol Sci. 2024;25(11):5820. doi: 10.3390/ijms25115820 38892007 PMC11172432

[27] Edera AA, Milone DH, Stegmayer G. Anc2vec: embedding gene ontology terms by preserving ancestors relationships. Brief Bioinform. 2022;23(2):bbac003. doi: 10.1093/bib/bbac003 35136916

[28] Szklarczyk D, Gable AL, Nastou KC, Lyon D, Kirsch R, Pyysalo S, et al. The STRING database in 2021: customizable protein-protein networks, and functional characterization of user-uploaded gene/measurement sets. Nucleic Acids Res. 2021;49(D1):D605–12. doi: 10.1093/nar/gkaa1074 33237311 PMC7779004

[29] Zitnik M, Rok Sosič, Leskovec J. BioSNAP Datasets: Stanford Biomedical Network Dataset Collection. 2018. http://snap.stanford.edu/biodata

[30] Liu T, Lin Y, Wen X, Jorissen RN, Gilson MK. BindingDB: a web-accessible database of experimentally determined protein-ligand binding affinities. Nucleic Acids Res. 2007;35(suppl_1):D198–201. doi: 10.1093/nar/gkl999 17145705 PMC1751547

[31] Hamilton W, Ying Z, Leskovec J. Inductive representation learning on large graphs. Adv Neural Inform Process Syst. 2017;30.

[32] Veličković P, Cucurull G, Casanova A, Romero A, Lio P, Bengio Y. Graph attention networks. arXiv preprint arXiv:171010903. 2017.

[33] Schölkopf B. Support vector learning. PhD Thesis. München, Germany: Oldenbourg; 1997.

[34] Breiman L. Random Forests. Mach Learn. 2001;45(1):5–32. doi: 10.1023/a:1010933404324

[35] Chen L, Tan X, Wang D, Zhong F, Liu X, Yang T, et al. TransformerCPI: improving compound-protein interaction prediction by sequence-based deep learning with self-attention mechanism and label reversal experiments. Bioinformatics. 2020;36(16):4406–14. doi: 10.1093/bioinformatics/btaa524 32428219

[36] Huang K, Xiao C, Glass LM, Sun J. MolTrans: Molecular Interaction Transformer for drug-target interaction prediction. Bioinformatics. 2021;37(6):830–6. doi: 10.1093/bioinformatics/btaa880 33070179 PMC8098026

[37] Bai P, Miljković F, John B, Lu H. Interpretable bilinear attention network with domain adaptation improves drug–target prediction. Nat Mach Intell. 2023;5(2):126–36. doi: 10.1038/s42256-022-00605-1

[38] Zeng X, Chen W, Lei B. CAT-DTI: cross-attention and Transformer network with domain adaptation for drug-target interaction prediction. BMC Bioinform. 2024;25(1):141. doi: 10.1186/s12859-024-05753-2 38566002 PMC11264959

[39] Abbasi K, Razzaghi P, Poso A, Amanlou M, Ghasemi JB, Masoudi-Nejad A. DeepCDA: deep cross-domain compound-protein affinity prediction through LSTM and convolutional neural networks. Bioinformatics. 2020;36(17):4633–42. doi: 10.1093/bioinformatics/btaa544 32462178

[40] Dehghan A, Razzaghi P, Abbasi K, Gharaghani S. TripletMultiDTI: Multimodal representation learning in drug-target interaction prediction with triplet loss function. Expert Syst Appl. 2023;232:120754. doi: 10.1016/j.eswa.2023.120754

[41] Wang H, Zhou G, Liu S, Jiang JY, Wang W. Drug-target interaction prediction with graph attention networks. arXiv preprint arXiv:210706099. 2021.

